# Investigation into a Lightweight Polymeric Porous Sponge with High Magnetic Field and Strain Sensitivity

**DOI:** 10.3390/nano12162762

**Published:** 2022-08-12

**Authors:** Yu Fu, Shijie Zhao, Zhenshuai Wan, Ye Tian, Shuangkun Wang

**Affiliations:** 1 School of Mechanical and Electrical Engineering, Henan University of Technology, Zhengzhou 450001, China; 2 School of Mechanics and Safety Engineering, Zhengzhou University, Zhengzhou 450001, China

**Keywords:** flexible sensors, polymeric porous sponge, mechanical properties, micron and nanoscale magnetic particles, electrical response, multi-sensing mode

## Abstract

Recently, flexible sensors have gained significant attention due to their potential applications in soft robotics and biomimetic intelligent devices. However, the successful production of favorable flexible sensors integrated with high flexibility, sensitivity and excellent environment adaptability toward multiple external stimuli is still an enormous challenge. Herein, a lightweight polymeric porous sponge capable of detecting an external magnetic field and strain excitations is proposed by assembling a sodium alginate/chitosan (SA/CHI) porous sponge with micron carbonyl iron and nanoscale Fe_3_O_4_ magnetic particles (MPs). Based on the double network structure, the SA/CHI sponge possesses preferable mechanical strength and hydrophilicity, demonstrating its high flexibility and deformability. More importantly, the electrical response of the SA/CHI sponge sensors can display remarkable variation under external magnetic and mechanical stimuli due to their superior magnetic characteristics and electrical conductivity. Meanwhile, their sensing properties can maintain relatively stable recoverability and repeatability towards the periodic excitations and releases. Additionally, a potential mechanism is provided to investigate their stimuli-sensitive behavior. It is highly dependent on the microstructure variations in MPs and conductive multi-walled carbon nanotube (MWCNTs) networks. Due to its exceptional magnetic controllability and appropriate electrical sensitivity, the proposed sensor shows high potential in wearable multi-sensing electronics and intelligent transport devices.

## 1. Introduction

Flexible sensing materials are a kind of functional composite which can efficiently detect and respond to external stimuli such as temperature [1,2], magnetic field [3,4,5], pressure [6,7], and humidity [8]. During the last few decades, owing to their high flexibility and unique stimuli-responsive performance, they have exhibited great potential in sensing systems, biomedical devices, wearable electronics skins, soft robotics, etc. [9,10,11,12]. Recently, with the epidemic spread of coronavirus disease 2019 (COVID-2019) around the world, an effective method to interrupt transmission is to avoid touching external objects. In this case, magnetic sensing materials have attracted increasing attention due to their advantages of contactless sensing capability, ideal remote controllability and environment adaptability [13]. Among them, magnetorheological (MR) materials are a type of flexible stimuli-responsive composite, in which soft magnetic particles (MPs) are embedded into the nonmagnetic polymeric matrix [14,15,16]. More importantly, their stimuli-responsive properties are capable of being adjusted rapidly and reversibly by an external magnetic field [17,18].

In recent years, a large number of functional materials based on MR materials have been developed and attempted to be employed in the field of intelligent sensing [19,20,21,22]. Nevertheless, these sensors are often used in sophisticated environments where various stimuli exist. Thus, it is necessary to devote further efforts to develop the functional sensors that can perceive different excitations in multi-field circumstances. In general, the responsive properties of MR materials-based sensors are highly dependent on the magnetic interactions among MPs, as well as the bonding forces of polymeric matrix. To meet the requirements of high magnetization and dispersion stability, we prepared bidisperse MPs consisting of micron carbonyl iron (CI) and nanoscale Fe_3_O_4_ particles [23]. By using a sol–gel method, gelatin (GE) and a multi-walled carbon nanotube (MWCNTs) were wrapped on the surface of MPs, which could produce flocculation and, consequently, enhance their inherent MR properties. Interestingly, due to the high conductivity of MWCNTs, the above core–shell structured MPs are available for multifunctional intelligent sensors.

On the other hand, the polymeric matrix plays an essential role in contributing to the long-term stability and stimuli-responsive properties of sensors [24]. Nowadays, varieties of high-performance matrix structures including fibers, thick film, multilayer sandwich as well as porous structures have been successively developed as sensing materials [25,26,27,28]. Ding et al. reported a multifunctional strain/magnetic sensor with superior flexibility, good sensitivity and conductivity, which enabled it to implement the real-time perception of external magnetic and strain stimuli [29]. Then, Shu et al. fabricated a magnetically responsive piezoresistive sensor composed of an MR elastomer, silver nanowires and flax fiber [30]. Based on high mechanic-electric-magnetic coupling performance, the cross-shaped sensor could be integrated into clothing for detecting and monitoring human motion. Additionally, Makarov et al. proposed a soft electronic skin equipped with tactile and contactless modes in a single sensor unit [31]. The finger-motion-correlated measurements indicated that the skins were expected to bring benefits for robotics and medical applications. Although the micro-nano structures exist on the surface of most matrices and are capable of sensing quite small magnetic field excitations, the sensors failed to satisfy the ongoing demand for detecting large magnetic fields and high sensitivity due to their limited deformation. Among them, the porous structures are highly desirable for the superiorities of high flexibility, low density and preferable deformability [32,33]. Moreover, the response performance could be greatly adjusted by varying the interior pore structures. Very recently, many functional polymers such as polyurethane [34,35], polydimethylsiloxane [36,37], silicone rubber [38] and natural biopolymers [39] have been introduced into the development of porous structures. In comparison to other polymers, sodium alginate (SA) and chitosan (CHI), possessing an ultra-low cost, structural diversity and fully biodegradability, are beneficial to constructing sensitive porous structures [40,41]. As natural biopolymers, they can successfully generate a physically crosslinked double network via the strong electrostatic interactions, which effectively improves the mechanical strength and structural stability of porous structures [42].

In this work, we reported a novel lightweight polymeric porous sponge towards the magnetic field and strain sensor by introducing MPs coated with MWCNTs into the SA/CHI porous sponge. The mechanical properties and electrical response under different external stimuli were systematically studied. The SA/CHI composite sponge exhibits preferable mechanical strength, excellent flexibility, deformability and appropriate sensing properties. Particularly, their electrical response can display remarkable variation under external magnetic and mechanical stimuli due to their superior magnetic characteristics and electrical conductivity. Meanwhile, their sensing properties can maintain a relatively stable recoverability and repeatability towards periodic excitations and releases. Moreover, a potential mechanism was provided and analyzed for a detailed insight into its stimuli-sensitive behavior. Based on its preferable magnetic controllability and electrical sensitivity, the SA/CHI porous sponge sensor exhibits effective and stable detection ability towards the external magnetic field and strain, which shows promising applications in future multi-functional sensing devices and wearable electronics.

## 2. Materials and Methods

### 2.1. Fabrication of SA/CHI Porous Sponge

**Figure 1:**
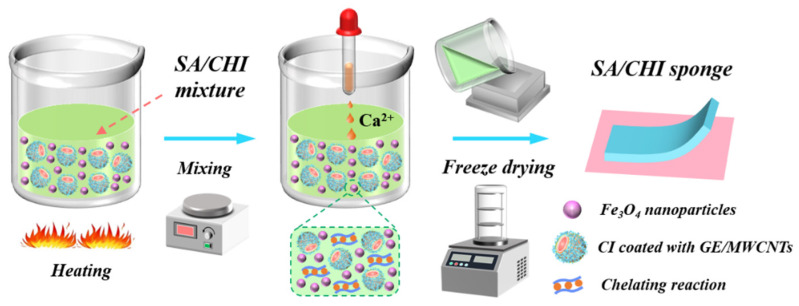
Fabrication procedures of the SA/CHI porous sponge.

The fabrication processes of the SA/CHI porous sponge are presented in Figure 1. Firstly, SA and carboxylation CHI powders with different concentrations (1–3 wt%) were dissolved into 25 mL of distilled water, respectively. Then, SA solutions and certain contents of glycerol were successively poured into the above CHI solutions and stirred at 70 °C for 1 h to ensure the uniform dispersion of the mixture. Afterwards, the bidisperse MPs containing CI (3.5 μm in average diameter, 7.9 g/cm^3^ in density) and Fe_3_O_4_ (20 nm in average diameter) particles dual-coated with GE/MWNCTs were added into above mixture, followed by homogeneously stirring for 2 h. Herein, the synthesis method of the bidisperse MPs could be obtained from our previous study [23]. Next, the slurry-like solution was coated with calcium chloride dispersion and heated in the water bath for another 30 min. After being treated with ultrasonic oscillation or vacuum drying for 30 min, the effect of bubbles was basically removed. Finally, the prepared suspension was poured into a dry square mold and pre-frozen at −60 °C for 8 h, followed by freeze-drying for 14 h, during which the synthetic composites were cured under the magnetization. After being demolded, this novel SA/CHI porous sponge was acquired.

Taking into account their mechanical strength, the samples with various SA/CHI mass ratios were prepared and labeled as SA1/CHI3, SA2/CHI3, SA3/CHI3, SA3/CHI2, and SA3/CHI1, respectively. Additionally, the glycerol contents in the samples were maintained at 0, 2, 4 and 6 wt%, respectively. In addition, the SA/CHI sponges treated with ultrasonic oscillation and vacuum drying were defined as SA/CHI-*M* and SA/CHI-*N*, respectively. The detailed parameters are shown in Table 1.

### 2.2. Characterization

The microstructures of MPs and the SA/CHI sponge were characterized using a scanning electron microscope (SEM, Gemini 500, Carl Zeiss Jena, Oberkochen, Germany). The chemical structure and synthesis mechanism were analyzed by Fourier transform infrared spectroscopy (FTIR, Spectrum 100, PerkinElmer, Waltham, MA, USA) from 4000 to 500 cm^−1^. Magnetization curves of various MPs were measured by a vibrating sample magnetometer (VSM, Zhengzhou University, Zhengzhou, China). The tensile mechanical properties and tensile strain-dependent electrical response were tested by a flexible device analysis system (AES-4SD, Beijing Zhongju High Technology Co., Ltd., Beijing, China). Each test was repeated five times. The required magnetic fields were supplied by a magnetic power system (SB-60, Changchun Yingpu Magneto-electric Technology Development Co., Ltd., Changchun, China).

The water absorption capability of SA/CHI sponge was investigated by swelling the dried samples in the distilled water at laboratory temperature, during which the samples were cut into the sheets of 10 mm× 10 mm× 3 mm. Additionally, the swollen samples were weighed after 2 h. Its water absorption was calculated by equation:

(1) $$R_{w}=\frac{M_{2}-M_{1}}{M_{1}}\times 100\%$$

where $M_{1}$ and $M_{2}$ represent the weights of SA/CHI sponge in the initial and stable state, respectively. The relative variation in electrical resistance was defined by [43]:

(2) $$\frac{\Delta R}{R_{0}}=\frac{\left|R-R_{0}\right|}{R_{0}}\times 100\%$$

where $R_{0}$ and *R* are the initial and measured resistances, ΔR is the resistance variation.

## 3. Results and Discussion

### 3.1. Structural Characterization of SA/CHI Porous Sponge

**Figure 2:**
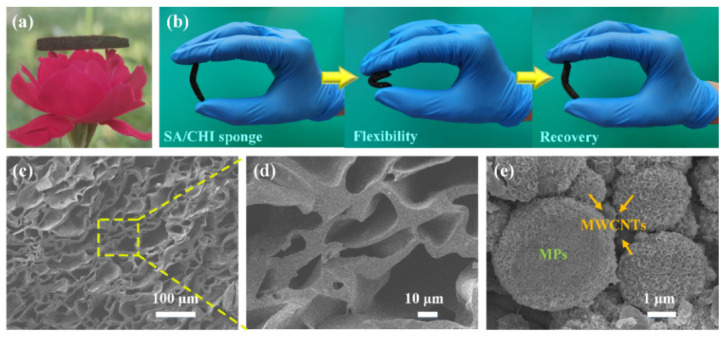
(**a**) The as-prepared SA3/CHI2 sponge put on the petals. (**b**) Flexibility and recoverability of SA3/CHI2 sponge. SEM images of (**c**,**d**) SA3/CHI2 sponge and (**e**) Micron CI and nanoscale Fe_3_O_4_ particles dual-coated with GE/MWCNTs.

The as-prepared product is shown in Figure 2a. The SA3/CHI2 porous sponge (25 mm× 5 mm× 3 mm) could be placed on petals without causing damage, displaying a lightweight advantage. Moreover, the sample could be deformed and totally recovered, which demonstrated that SA/CHI porous sponge has excellent flexibility and recovery capabilities (Figure 2b). Figure 2c,d shows the microstructure of SA3/CHI2 sponge under different magnifications. It was found that the distribution of pores was uniform, and the MPs containing micron CI and nanoscale Fe_3_O_4_ were well dispersed in the pore walls. The MPs showed a clear core–shell structure, the surface of which were dual-coated with GE/MWNCTs (Figure 2e). The quite rough surfaces could obviously improve the dispersion stability and MR properties of MPs.

**Figure 3:**
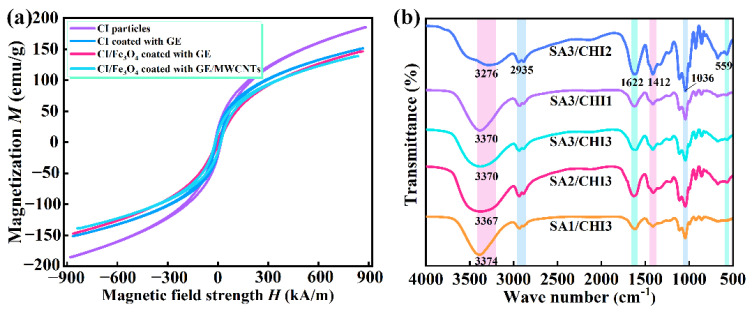
(**a**) Magnetic hysteresis loops of different MPs. (**b**) FTIR spectrum of SA/CHI porous sponge.

The magnetic property plays an important role in determining magnetic sensitivity of SA/CHI porous sponge. The intrinsic magnetic properties of different MPs were investigated at room temperature. The saturation magnetization for CI, CI coated with GE, and CI/Fe_3_O_4_ coated with GE particles were 185, 151 and 147 emu/g, respectively (Figure 3a). Due to the coating of MWCNTs, its saturation magnetization slightly decreased to 144 emu/g, which indicated that the GE/MWCNTs composite layer did not affect the inherent magnetic properties. Furthermore, the introductions of Fe_3_O_4_ nanoparticles decreased the overall coercivity of bidisperse MPs, thus resulting in the preferable magnetic properties CI/Fe_3_O_4_ wrapped with GE/MWCNTs. Clearly, the SA/CHI sponge exhibits typical soft magnetic properties. Figure 3b shows the FIIR spectrum of SA/CHI sponges in the range of 4000–500 cm^−1^. By comparison, the vibration peaks of O-H at 3400–3200 cm^−1^ continuously migrated for various samples, suggesting that the SA interacted with CHI, and the hydrogen bonds formed by polymers and water molecules were altered. Taking the SA3/CHI2 sponge as an example, the asymmetric and symmetric stretching vibrations of –COO^-^ were observed at 1622 and 1412 cm^−1^, respectively. Among them, the variation in the vibration absorption peak at 1622 cm^−1^ arose from the chelating reaction between SA and Ca^2+^ and the electrostatic action between SA and CHI. The band located at 1036 cm^−1^ indicated the absorption peak of C-O. Additionally, the characteristic Fe-O peak was near 559 cm^−1^, which clearly showed the bonding interactions between MPs and GE protein molecules. In summary, the synthesis mechanism of the SA/CHI sponge is illustrated: SA is an anionic polyelectrolyte with many –COOH, and CHI is a cationic polyelectrolyte with a large number of –NH_2_ on the molecular chain [44]. They can generate a composite hydrogel structure through the biological crosslinking reaction. Meanwhile, the multiple oxygen atoms on the G-block of SA were capable of generating a three dimensional “egg-box” structure with Ca^2+^, which is the so-called chelating reaction. These two reactions occurred independently and simultaneously, contributing to the formation of a physically crosslinked double-network. So, the SA/CHI porous sponge possesses the ideal mechanical strength and structural stability.

**Figure 4:**
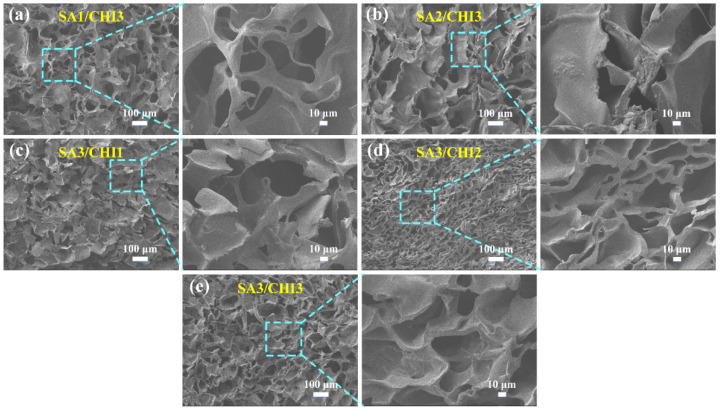
Cross-sectional views of different SA/CHI porous sponges in different magnifications. (**a**) SA1/CHI3. (**b**) SA2/CHI3. (**c**) SA3/CHI1. (**d**) SA3/CHI2. (**e**) SA3/CHI3.

The morphologies of SA/CHI porous sponge with various SA/CHI mass ratios are observed in Figure 4 in detail. All the samples comprised a large number of MPs, pore walls, and pores with different average diameters. With the increase in SA, the thickness of the pore walls improved while the quantity of pores reduced. Simultaneously, the thickness of the pore walls and pore sizes obviously increased with the augments of CHI contents. It showed that sufficient electrostatic interactions significantly enhanced the crosslinking density of polymers, thereby reducing their crystalline phase and improving the uniformity and compactness of the network structures. So, the SA3/CHI3 sponge possesses the most uniform pores and structural stability. However, redundant SA and CHI contents may lead to excessive film thickness and the fewer amounts of pores reduced its inherent flexibility. By comparison, the SA3/CHI2 sponge was selected for further investigations.

**Figure 5:**
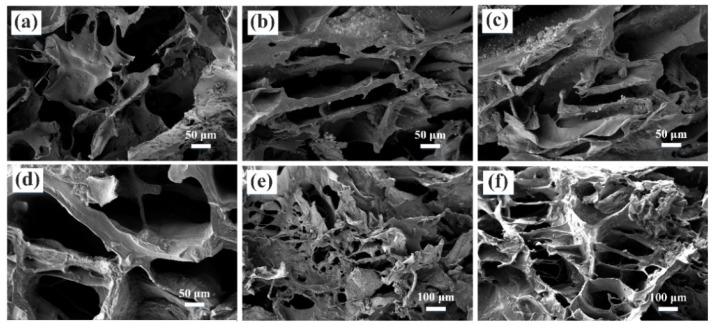
Cross-sectional views of different SA/CHI porous sponges. (**a**) SA3/CHI2-0. (**b**) SA3/CHI2. (**c**) SA3/CHI2-4. (**d**) SA3/CHI2-6. (**e**) SA3/CHI2-*M*. (**f**) SA3/CHI2-*N*.

The effect of glycerol on the microstructures is illustrated in Figure 5a–d. As a moisturizer and plasticizer, the introduction of glycerol is capable of adjusting the pore walls thickness and flexibility of prepared samples. Obviously, the thickness of the pore wall increased, and the pore structures were more evenly distributed with the glycerol contents, varying from 0 to 6 wt%. Nevertheless, the excessive glycerol may greatly enlarge the pore size and loosen their network structures, hence affecting the viscosity and compactness of the sponges. On the other hand, Figure 5e,f shows the cross-sectional views of SA3/CHI2-*M* and SA3/CHI2-*N*, respectively. It was found that the distribution of cavities was more homogeneous with the help of ultrasonic oscillation processing. Because the medium molecules of samples may be squeezed and discretized by the ultrasonic waves, leading to the formation of bubbles or cavities. These cavities gradually increased and eventually became unstable and collapsed, thereby releasing high temperature and pressure. On this basis, the adoption of freeze-drying technology made it possible to generate a more compact porous sponge.

### 3.2. Mechanical Property and Hydrophilicity of SA/CHI Porous Sponge

**Figure 6:**
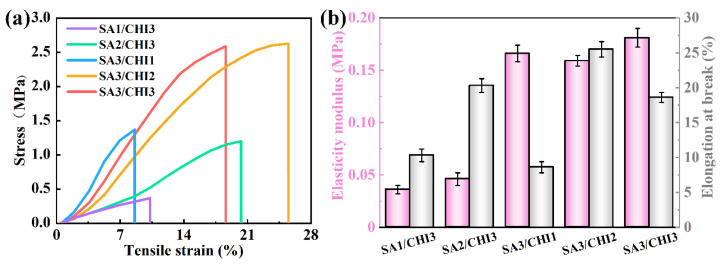
(**a**) Tensile stress/strain curves for various samples. (**b**) Test results of elasticity modulus and elongation at break for various samples.

Tensile tests were employed to study the mechanical properties of SA/CHI porous sponges. Figure 6a shows the tensile behaviors for different samples, respectively. In the small strain level, the tensile stress was nearly linear. Without loss of generality, they all exhibited an elastic stage and eventually fracture. Then, their elasticity modulus and elongation at break were analyzed and recorded, as shown in Figure 6b. It was obvious that the tensile strength gradually increased from 0.036 to 0.181 MPa with the mass fraction of SA varying from 1 to 3 wt%, which is mainly due to the continuous improvement of the film thickness. Meanwhile, as CHI contents increased, the elongation at break was greatly raised, thus resulting in a better flexibility, whereas excessive CHI reduced the elongation at break of the samples. This phenomenon may ascribe to the decrease in their flexibility and deformability. In comparison to other samples, the SA3/CHI3 sponge possesses the highest elasticity modulus of 0.181 MPa, while the SA3/CHI2 sponge has the preferable elongation of 25.5% at break under the external stress or strain.

**Figure 7:**
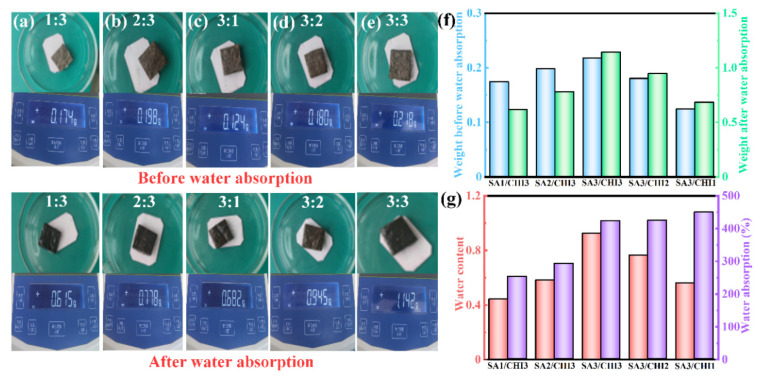
(**a**–**e**) Photographs of various SA/CHI sponges before and after water absorption. (**f**,**g**) Weight and water absorption ratio $R_{w}$ for various samples, respectively.

The hydrophilicity also plays an essential role in determining the porosity and water absorption capacity of the SA/CHI porous sponges. Figure 7a–e shows the digital images of the water absorption test for different sponge samples in the initial and stable stage, respectively. Their corresponding results are recorded and calculated in Figure 7f,g. With the increase in SA, the water absorption of SA/CHI sponges displayed an upward trend. Specifically, the water absorption reached the maximum when the SA content was 3 wt%. There were numerous monomers in the structures that were not involved in the reaction when the SA content was fewer, which was unfavorable for the synthesis of three-dimensional network, thus resulting in a lower porosity. Moreover, the addition of SA containing a large number of –COOH and –OH could introduce many strong hydrophilic groups that were capable of binding with water molecules into the hydrogen bonds, so the water absorption capacity of SA/CHI sponges was significantly improved. Importantly, the water absorption contents could reach 2.53, 2.93, 7.45, 4.25, and 2.56 times their own weights for samples SA1/CHI3, SA2/CHI3, SA3/CHI3, SA3/CHI2, and SA3/CHI1, respectively. The excellent water absorption ability was closely linked to the structural morphology and processing technology of the films. The surface on the films fabricated by natural or vacuum drying technology showed some wrinkled appearances, and these relatively dense and inferior permeability films affected the entry of water molecules, which greatly limited their water absorption capability. Nevertheless, the films acquired by freeze-drying technology had a uniform and broad pore structure. The water molecules could be fully diffused into the pores, so the water absorption was preferable. Ideal water absorption capability can ensure the stability and elastic expansion of the polymer molecular chain, maintaining the free transmission of the –OH group in the water molecules. Owing to the above outstanding characteristics, the SA/CHI sponges were capable of acquiring superior mechanical properties and deformability. In summary, it is accepted that the sample SA3/CHI2 possesses excellent flexibility, deformability and high mechanical strength.

### 3.3. Magnetic Field and Strain-Dependent Electrical Properties of SA/CHI Porous Sponge

**Figure 8:**
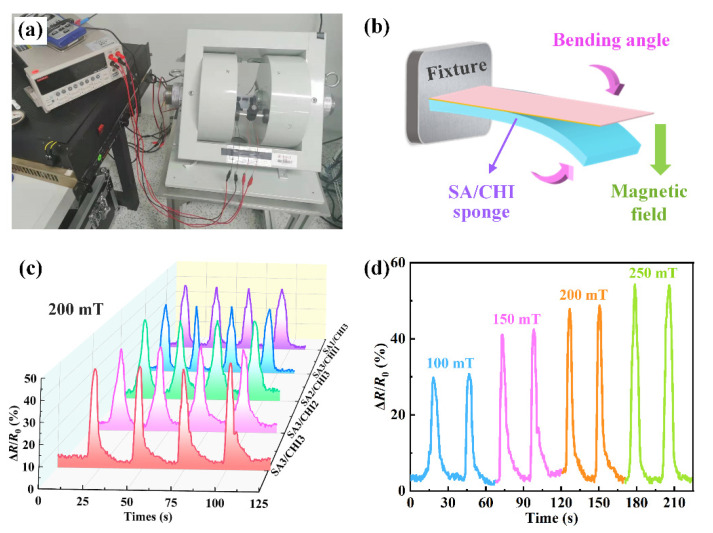
(**a**) Photographs of the experimental setup in the magnetic responsive performance test. (**b**) Schematic illustration of magnetic responsive behavior for SA/CHI sponge. (**c**) Cyclic tests of relative resistance variation for different SA/CHI sponge sensors under the magnetic field of 200 mT. (**d**) Relative resistance variation from 100 to 250 mT for SA3/CHI2 sponge sensor.

Owing to the incorporated bidisperse MPs, the magnetic response performance of SA/CHI sponge was necessarily investigated. Figure 8a,b shows the experimental setup and schematic diagram of magnetic field-dependent electrical response tests. Herein, the samples could bend to various angles and recover when the external magnetic field force was applied and withdrawn. Importantly, thanks to the particle interactions among MPs embedded into the SA/CHI sponge, the samples possess exceptional magnetic characteristics, and can be employed as a lightweight magnetic field sensor.

By applying a cyclic magnetic field of 200 mT, the peak values of $\Delta R/R_{0}$ were maintained at around 47.8%, 45.4%, 44.7%, 39.8%, and 38.9% for samples SA3/CHI3, SA3/CHI2, SA2/CHI3, SA3/CHI1, and SA1/CHI3, respectively, proving their good repeatability and electrical response capability (Figure 8c). Next, the SA3/CHI2 sponge-based sensor was selected for further studies. By varying the magnetic field amplitude from 100 to 150, 200 and 250 mT, the peak values of $\Delta R/R_{0}$ obviously increased from 30.8% to 40.5%, 45.7% and 52.8% (Figure 8d). The higher the external magnetic fields, the larger the bending deformations and electrical resistance variations, which further demonstrated its superior deformation stability and magnetic sensitivity. It is attributed to the fact that with increasing of external magnetic fields, the magnetic interactions formed by the bidisperse MPs were reinforced, hence leading to the compactness of MPs chains and apparent changes in resistance properties. Therefore, the SA3/CHI2 sponge sensor is suitable for contactless magnetic field monitoring.

**Figure 9:**
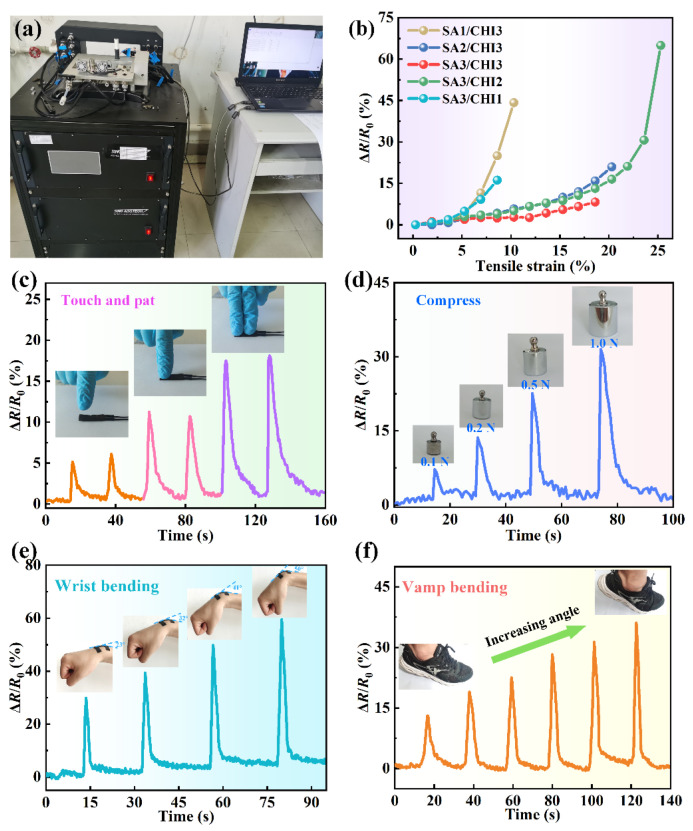
(**a**) Photographs of the experimental setup in the strain responsive performance test. (**b**) Relative resistance variation as a function of tensile strain for different SA/CHI sponges. Photographs and relative resistance variation along with time of the SA3/CHI2 sponge sensor excited by (**c**) slight touch and pat, (**d**) compression with different weights, (**e**) wrist bending and (**f**) vamp bending.

Based on the effect of conductive networks formed by MWCNTs, the assembled SA/CHI composite sponge presents outstanding electrical sensitivity toward multiple mechanical stimuli. To investigate the stretch-dependent electrical properties of SA/CHI sponges, they were fixed on the experimental setup in which the electrical responses could be simultaneously recorded (Figure 9a). Figure 9b shows the $\Delta R/R_{0}$ as a function of tensile strain for various SA/CHI sponges. The $\Delta R/R_{0}$ increased along with the augment of tensile strains, which varied from 0 to 25.5%. Importantly, destruction could occur after all the samples reached their corresponding maximum strain during the test, then the acquisitions of electrical response would be continued. It could be observed that the variation process of $\Delta R/R_{0}$ mainly consisted of slowing and quickly changing stages. Moreover, the maximum $\Delta R/R_{0}$ was 44.2%, 21.0%, 8.24%, 64.9%, and 16.2% for samples SA1/CHI3, SA2/CHI3, SA3/CHI3, SA3/CHI2, and SA3/CHI1, respectively. Clearly, the sample SA3/CHI2 sponge exhibited preferable stretch-responsive capability, which caused its selection for further compression measurements.

The results of electric response toward various compression stimuli are presented in Figure 9c. When stimulated by slight touch and pat, the SA3/CHI2 sponge sensor was able to respond immediately and recovered to the initial state, which illustrated its preeminent response sensitivity and reliable stability. Furthermore, during the patting process with different fingers, its electric response showed obvious differences, so the sensing performance was closely related to the active contact areas. Finally, Figure 9d shows the electric response of SA3/CHI2 sponge sensor stimulated by compressive stresses. The Δ*R*/*R*_0_ was significantly varied with the compression of different external pressures, which indicated its good sensing capability and recoverability. More importantly, the sponge sensor was capable to be tightly attached to the surface of human skin. When the wrist gradually bent, the sensor produced the bending deformation, as shown in Figure 9e. The maximum $\Delta R/R_{0}$ varied from 29.9% to 59.5% once the angles augmented from 23° to 50°. Moreover, the sponge sensor also performed well in the perception of vamp bending (Figure 9f). In this case, with the continuous increase in bending angles, larger electrical responses were obtained. Undoubtedly, it shows superior stability and responsiveness for the real-time detection of continuous human joint movements.

In conclusion, the as-prepared SA3/CHI2 sponge presents exceptional sensitivity and reliable stability under magnetic fields, stretching, compressing, and bending strains, which shows its promising applications in wearable electronics and integrated sensing devices.

### 3.4. Stimuli-Sensitive Mechanism of SA/CHI Porous Sponge

**Figure 10:**
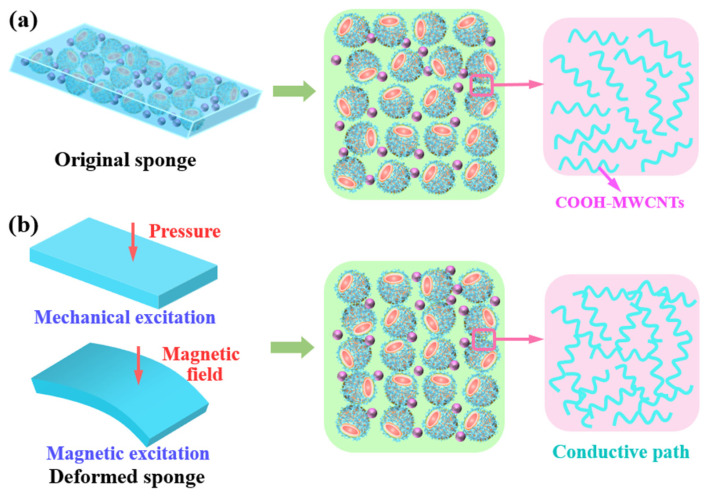
Schematic illustration of the stimuli-sensitive mechanism. (**a**) Microstructure of sample and conductive MWCNTs networks in the initial state. (**b**) Deformation of sample and conductive MWCNTs networks under various stimuli.

In this work, the stimuli-sensitive mechanism of SA/CHI porous sponge is mostly dependent on the microstructure variation of bidisperse MPs and conductive MWCNTs networks. To further demonstrate its sensitivity properties, the response behaviors toward various external stimulations are presented in Figure 10. SA/CHI sponge is typical magnetically responsive material, which contains a large number of CI, Fe_3_O_4_ particles and pore structures. The nanoscale Fe_3_O_4_ particles are arbitrarily attached and filled into the interspaces formed micron CI particles, which can significantly change the interactions among CI particles through the Brownian motion of Fe_3_O_4_ particles, consequently resulting in an enhanced disperse stability of MPs. In addition, their micro-nano structures generally exist on the surface of the matrix and generate deformations to alter their internal contact resistance or dielectric coefficient, thereby realizing the detection of external stimuli. Initially, these MPs wrapped with GE/MWCNTs are randomly distributed in the porous structures (Figure 10a). Under applying an external magnetic field or pressure, the MPs are gathered together, resulting in the increase in effective contacting areas and conductive paths formed by the MWCNTs coatings, which contributes to the promotion of contact resistance for the SA/CHI sponge. On the other hand, the action of magnetic and mechanical forces obviously adjusts the shape, volume of sponges, and the distribution of conductive MWCNTs, which further induces the variation in its conductivity and sensitivity. As a result, SA/CHI porous sponge could serve as a sensor to percept the external magnetic field and mechanical excitations.

## 4. Conclusions

In this paper, a novel lightweight SA/CHI sensor was developed by embedding bidisperse MPs coated with GE/MWCNTs in the SA/CHI sponge. By constructing a double network structure, SA/CHI sponge possesses preferable mechanical strength and hydrophilicity, demonstrating its high flexibility and deformability. Interestingly, based on its superior magnetic characteristics, the SA/CHI sponge sensor could detect various magnetic field strengths in contactless mode. For instance, the maximum electrical response of the SA3/CHI2 sponge sensor varied from 30.8% to 52.8% when the magnetic fields increased from 100 to 250 mT. Moreover, the SA/CHI sensor shows excellent sensing performance under mechanical stimuli such as stretching, compressing and bending. In particular, its electrical response could maintain superior stability and repeatability under the effect of periodic external magnetic and mechanical stimuli, which demonstrated that it could be employed as an intelligent magnetic field/strain sensor towards multiple environments. Since its resistance properties show strain-dependent characteristics, it can also monitor different human movements. In addition, a possible mechanism was provided to explore the stimuli-sensitive behavior of SA/CHI porous sponge. It is highly dependent on the microstructure variation of bidisperse MPs and conductive MWCNTs networks. In conclusion, the SA/CHI porous sponge sensor with stable magnetic/strain sensing capability could exhibit great potentiality in future intelligent wearable devices and soft robotics.

## Figures and Tables

**Table 1:** Detailed parameters of SA/CHI porous sponge.

Samples	Mass Fraction (wt%)		Glycerol (wt%)	Ultrasonic Oscillation (h)	Vacuum Drying (h)
	SA	CHI			
SA1/CHI3	1	3	2	0	0
SA2/CHI3	2	3	2	0	0
SA3/CHI3	3	3	2	0	0
SA3/CHI2	3	2	2	0	0
SA3/CHI1	3	1	2	0	0
SA3/CHI2-0	3	2	0	0	0
SA3/CHI2-4	3	2	4	0	0
SA3/CHI2-6	3	2	6	0	0
SA3/CHI2-*M*	3	2	2	0.5	0
SA3/CHI2-*N*	3	2	2	0	0.5

## Data Availability

The data presented in this study are available on request from the corresponding author.
